# Predictive factors of axillary lymph node involvement in Tunisian women with early breast cancer

**DOI:** 10.4314/ahs.v23i4.30

**Published:** 2023-12

**Authors:** Leila Achouri, Ines Zemni, Amani Jallali, Houyem Mansouri, Najet Mahjoub, Houda Henchiri

**Affiliations:** 1 Jendouba hospital, Oncologic surgery; 2 Institut Salah-Azaiz, Department of surgical oncology; 3 Jendouba Hospital, Department of medical oncology

**Keywords:** early breast cancer, predictors, axillary lymph node

## Abstract

**Background:**

Axillary lymph node involvement (ALNI) is associated with an increased risk of local recurrence and poor prognosis in early breast cancer. The determination of the risk of positive axillary lymph node contributes to therapeutic decisions.

**Objectives:**

The aim of this study was to identify clinicopathological predictive factors of axillary lymph node metastases in patients with early breast cancer.

**Methods:**

We included patients with clinical T0, T1 andT2 invasive breast carcinoma who underwent resection of the primary tumor and axillary staging by sentinel lymph node biopsy and/or axillar lymph node dissection between 2012 and 2018.

**Results:**

Of the 135patients included, 41.5% had ALNI. Regarding univariate analysis, clinical factors correlated with positive ALNM were clinical tumour size>30mm, clinical tumour stage, clinical number of tumours, clinical axillary nodal status and nodal status on ultrasound. Pathologic factors associated with nodal involvement were pathologic tumour stage, tumour grade SBR, number of foci, lymphovascular invasion, perineural invasion and Ki67>20%.

In multivariate logistic regression, clinical axillary nodal status, pathologic tumour stage and lymphovascular invasion (LVI) remained as independent predictors of ALNI.

**Conclusions:**

Based on these results, we suggest that clinical axillary nodal status, pathologic tumour stage and LVI are predictive factors for ALNM in Tunisian women with early breast cancer.

## Introduction

Axillary lymph node involvement is associated with an increased risk of local recurrence and poor prognosis in early breast cancer. The nodal status also determines the need for adjuvant therapy 1. The physical examination of axillary lymph nodes is relatively simple to perform, but it is inaccurate with up to 60% false negativity 2.

Therefore, axillary lymph node dissection (ALND) has been the standard of care, in order to provide accurate staging and loco regional disease control 3. On the other hand, it is associated with a significant morbidity, such as lymphedema and functional limitations of the affected upper limb 4.

However, sentinel lymph node biopsy (SLNB) is currently a suitable alternative to ALND and is the standard treatment for axillary staging in clinically node negative patients, while avoiding the morbidity of the latter 5,6.

SLNB requires a multidisciplinary team, including surgeons, radiologists, nuclear medicine specialists and pathologists, which is not always available in some hospitals. Nevertheless, the determination of the risk of positive axillary lymph node can significantly contribute to therapeutic decisions 7. In fact, the identification of predictive factors of axillary lymph node metastases would be effective in sparing axillary lymph node surgery and reducing subsequent complications 8.

In Tunisia, breast cancer is more frequently diagnosed in an advanced stage, with a higher proportion of young women, compared to international series 9. The aim of this study was to identify clinicopathological predictive factors of axillary lymph node metastases in patients with early invasive breast cancer in the north west of Tunisia.

## Methods

Epidemiological, clinical, operative and pathological data for all breast cancer patients undergoing breast cancer surgery at the department of oncologic surgery, regional hospital of Jendouba, were collected. After institutional review board approval, we retrospectively reviewed all cases in this database between January 2012 and December 2018.

We included patients with clinical T0, T1 and T2 (AJCC, 8th edition) 10 invasive breast carcinoma who underwent resection of the primary tumor and axillary staging by SLNB and/or ALND. Patients treated for metastatic disease, carcinoma in situ, local recurrence or those who received neoadjuvant treatment were excluded from the study. Breast cancer patients seen in our institution but who did not receive surgery were not included in this study.

Clinical factors evaluated were: age at diagnosis, parity, menopausal status, family history of breast cancer, clinical tumour size, clinical number of tumours, tumour location, clinical and radiological axillary lymph node involvement, and radiological features of the mass evaluated according to the Brest Imaging Reporting and Data System (BI-RADS) of the American College of Radiology (ACR) 11.

Pathological factors assessed included tumour size, histologic grade (modified Bloom and Richardson system: SBR), histologic subtype, number of foci, lymphovascular invasion, perineural invasion, progesterone and oestrogen receptor status (studied by immunohistochemical analysis and recorded as negative or positive), human epidermal growth factor receptor Her-2, Ki 67 and histologic axillary involvement.

In the cases where patients had multiple foci, the size and location of the largest focus were used for the analysis. Based on immunohistochemical (IHC) analysis, positivity for estrogen receptor (ER) and progesterone receptor (PR) was defined as nuclear staining of >10% of tumor cells. Regarding the HER2 status, it was evaluated by IHC and confirmed by chromogenic in situ hybridization (CISH) in case of HER2 2+. For the expression level of the Ki67 index, Ki67≤20% was regarded as low expression, and >20% was regarded as high expression. Five molecular subtypes were defined according to clinico-pathological criteria 12.

All data were analysed using the SPSS statistical software package (version 21). Continuous normally distributed variables are expressed by their mean and standard deviation. Not normally distributed variables are expressed as medians, and their interquartile (IQR) ranges and categorical variables are expressed as n (%). To compare the continuous variables with normal distribution, we used the t-test. In case of not normally distributed, we used the Mann–Whitney test. To compare the categorical variables, we used chi-square or Fisher test if the assumption for the first was not complied.

For the estimation of risk in the multivariate analysis, we used the logistic regression, expressing the odds ratio (OR) and 95% confidence interval (CI). Statistical significance was considered to be at p < 0.05.

## Results

A total of 135 patients met the inclusion criteria. Clinical patients' characteristics are summarized in Table1. Patients' mean age was 52.09 years +- 13.38, ranging from 24 to 90 years. The median clinical tumour size was 25mm (Q25 :20, Q75 :30). Most of the patients had T2 tumours (51.9%) and only six patients had T0 tumour (4.4%). Almost all patients had axillary lymph node dissection, only three patients had sentinel lymph node biopsy.

**Table 1:** Clinical patients characteristics

Variable	Number of patients	Percentage (%)
**Age (in years)**		
≤ 50	69	51.1
>50	66	48.9
**Menstrual status**		
Premenopausal	71	52.6
Postmenopausal	64	47.4
**Family history of breast cancer**		
Yes	12	8.9
No	123	90.1
**Clinical tumour size**		
≤30mm	100	78.5
>30mm	29	21.5
**Clinical tumour stage**		
T0	6	4.4
T1	59	43.7
T2	70	51.9
**Clinical Multifocality**		
Unifocal	123	91.1
Multifocal (≥2)	6	8.9
**Clinical tumour location**		
Lateral	84	65.1
Medial	22	17.1
Retroareolar	6	4.6
Overlapping	17	13.2
**Clinical axillary nodal status**		
Positive (cN1)	61	45.2
Negative (cN0)	74	54.8
**Nodal status on ultrasound**		
Positive	37	27.4
Negative	98	72.6
ACR* classification		
ACR3	3	2.2
ACR4	49	36.3
ACR5	83	61.5
**Type of surgery**		
Conservative	89	65.9
Radical	46	34.1

* ACR: American College of Radiology

The median pathologic tumour size was 20mm (Q25 :15, Q75 :25). The majority of patients (68.2%) had low grade carcinoma (grade 1 or 2). Invasive ductal carcinoma was the predominant tumor type (91.1%). The Ki67 value was mentioned in 96 cases, and the Her status in 131 cases. As a result, the molecular classification was possible for 113 patients. The hormone receptors ER/PR were positive in 74.8% of cases and 21.4% exhibited HER2 overexpression. Of the 135 patients, 56 (41.5%) were found to be node positive, and the mean number of nodes examined was 17.05, with a range from 1 to 40. Table 2 shows pathological characteristics of the patients' tumours.

**Table 2:** Pathological tumours characteristics

Variable	Number of patients	Percentage (%)
**Pathologic tumour stage**		
pT1	78	57.8
pT2	57	42.2
**Tumour grade SBR**		
1/2	92	68.2
3	43	31.8
**Histologic subtype**		
Ductal	123	91.2
Lobular	1	0.7
Other*	11	8.1
**Number of foci**		
1	111	82.2
≥2	24	17.8
**Lymphovascular invasion**		
Present	25	18.5
Absent	110	81.5
**Perineural invasion**		
Present	13	9.6
Absent	122	122
ER* status		
Positive	99	73.3
Negative	36	26.7
PR* status		
Positive	89	65.9
Negative	46	34.1
**Hormonal receptors**		
Negative	34	25.2
Positive	101	74.8
**Ki67**		
≤20%	45	46.9
>20%	51	53.1
**Her2neu**		
Overexpressed	28	21.4
Not overexpressed	103	78.6
**Tumour molecular subtypes**		
Luminal A	25	22.1
Luminal B Her-	46	40.7
Luminal B Her+	10	8.8
Her2 enriched	17	15
Triple negative	15	13.3
**Pathologic nodal status**		
Negative (pN0)	79	58.5
Positive (pN1)	56	41.5

*Other: medullary, mucinous, metaplastic *ER: estrogen receptor *PR: progesterone receptor

Regarding univariate analysis of factors associated with ALNM (Table3), clinical factors correlated with positive ALNM were clinical tumour size >30mm (p=0.006), clinical tumour stage (p=0.047), clinical number of tumours (p=0.016), clinical axillary nodal status (p<0.001) and nodal status on ultrasound (p<0.001). Pathologic factors associated with nodal involvement in univariate analysis were pathologic tumour stage (p=0.003), tumor grade SBR (p=0.001), number of foci (p<0.001), lymphovascular invasion (p<0.001), perineural invasion (p=0.001) and Ki67 >20% (p=0.049).

**Table 3:** Univariate analysis of factors associated with axillary lymph node metastasis

Factor	Node-Negative (n=79)	Node-Positive (n=56)	P Value
**Age (in years)**			
≤ 50	45	24	0.106
>50	34	32	
**Menstrual status**			
Premenopausal	46	25	0.119
Postmenopausal	33	31	
**Family history of breast cancer**			
Yes			
No	5	7	0.235
	74	49	
**Clinical tumour size**			
≤30mm	63	37	0.006
>30mm	10	19	
**Clinical tumour stage**			
T0	6	0	0.047
T1	37	22	
T2	36	34	
**Clinical number of tumours**			
Unifocal			
Multifocal	75	46	0.016
	4	10	
**Clinical tumour location**			
Lateral	44	40	0.519
Medial	13	9	
Retroareolar	4	2	
Overlapping	12	5	
**Clinical axillary nodal status**			
Positive (cN1)	23	38	
Negative (cN0)	56	18	<0.001
**Nodal status on ultrasound**			
Positive	12	25	
Negative	67	31	<0.001
**ACR**			
ACR3	3	0	0.246
ACR4	31	18	
ACR5	45	38	
**Pathologic tumour stage**			
pT1	54	24	0.003
pT2	25	32	
**SBR**			
1/2	63	29	0.001
3	16	27	
**Histologic subtype**			
Ductal	73	50	0.622
Lobular	1	0	
Other*	5	6	
**Number of foci**			
1	73	38	<0.001
≥2	6	18	
**Lymphovascular invasion**			
Present	6	19	
Absent	73	37	<0.001
**Perineural invasion**			
Present	2	11	0.001
Absent	77	45	
ER* status			
Positive	61	38	0.226
Negative	18	8	
PR* status			
Positive	54	35	0.479
Negative	25	21	
**Ki67**			
≤20%	31	14	0.049
>20%	25	26	
**Her2neu**			
Overexpressed	14	14	0.332
Not overexpressed	62	41	
**Tumour molecular subtypes**			
Luminal A	19	6	
Luminal B Her-	26	20	0.352
Luminal B Her+	6	4	
Her2 enriched	8	9	
Triple negative	8	7	

*Other: medullary, mucinous, metaplastic *ER: estrogen receptor *PR: progesterone receptor

Subsequently, multivariate logistic regression was carried out on those variables found to be statistically significant on univariate analysis. The results are presented in Table 4. The clinical axillary nodal status (OR=4.31, CI 2.26-50, p=0.032), the pathologic tumor stage (OR=3.66, CI 2-19.23, p=0.016) and the lymphovascular invasion (OR=4.29, CI 1.91-29.41, p=0.026) remained as independent predictors of axillary lymph node involvement.

**Table 4:** Multivariate analysis of factors associated with axillary lymph node metastasis

Variable	OR*	95% CI*	p-value
Clinical axillary nodal status	4.31	2.26 - 50	0.032
Pathologic tumour stage (pT)	3.66	2 – 19.23	0.016
LVI	4.29	1.91 – 29.41	0.026

OR: odds ratio, CI: confidence interval

## Discussion

In this study, the clinical axillary nodal status, the pathologic tumour stage and the lymphovascular invasion were independent factors of axillary lymph node involvement. These results are partially in concordance with other series published in the literature.

The overall incidence of ALNM in our series was 41.5%, which is slightly higher than previous researches from other populations, ranging from 33.2% to 41%. 7,8,13–15.

Lymphovascular invasion has been described as the strongest predictive factor of nodal involvement 8,13,15–19. LVI was also an independent factor of ALNM in our study. It could be regarded as a perquisite for the dissemination via the lymphatic 20. Some authors are even convinced that all tumours with nodal involvement have lymphatic invasion, detected by the pathologist or not 8. In fact, LVI was associated with a significant decrease in survival at 12-year follow-up in the series of Woo et al, despite the absence of nodal disease 21.

We showed an association between pathologic tumour stage and axillary metastasis, concurring with data from several other centers 2,8,13,19,22,23. The reported incidence of ALNM ranged from 21 to 42% for T1 tumours and 31 to 63% for T2 tumours 14,17,24, which is in accordance with our results (30.7% for T1 and 56.1% for T2 tumours). In deed, the larger is the tumor size, the higher is the probability of positive lymph nodes 13. However, small primary tumours with extensive lymph node metastases have been reported 25.

Despite our study suggested no significant association between ER/PR Her2 status and lymph node involvement, molecular markers have been widely evaluated and various studies have assessed the role of ER and PR receptors status in predicting axillary lymph node metastasis with initial conflicting results. In their study of 1416 early breast cancer patients, Capdet et al found no association between hormonal status and nodal positivity 17. However, more recent studies concluded that negative hormonal status had a reduced risk of axillary lymph node involvement compared to other patients, when adjusting for other risk factors 8,18,26.

Her2 negative tumours were found to be associated with lymph node involvement less frequently than Her 2 positive tumours 7.

The frequency of ALNM was higher in patients with Ki67 index >20% in the series of Chung et al, as well as our series 2.

Regarding molecular subtypes, the negative ER and negative Her2 tumours was associated with the lowest probability of node metastasis 7,27. In contrast, negative hormonal receptors and Her2 positive tumours had the highest probability 7. However, in their nomogram, Zong et al showed that early breast cancer with a higher rate of ALNM had a Luminal B-like subtype 1.

The number of foci has also been evaluated as a potential predictive factor. In our study, it did not retain in multivariate analysis. However, Yoshihara et al found that the number of foci of the primary tumour was a significant independent tumour 13.

The tumour location was rarely evaluated, and authors found a lower frequency of lymph node evolvement in the medial quadrant located tumours 13,28.

To the best of our knowledge, this is the first study that evaluates incidence and predictive factors of ALNM in a population of the north west of Tunisia, with early breast cancer. These results can help us to take specific therapeutic decisions in specific clinical situations concerning the management of the axilla in early breast cancer, especially when general anesthesia is contraindicated, in patients with unexpected diagnosis of invasive tumour or when an autologous breast reconstruction is indicated.

However, we need a reliable predictive model of lymph node positivity to be allowed to omit axillary dissection. Some authors already suggested that even SLNB could be omitted in tumours with good prognosis subtypes 7,29,30. Above all, these patients with favourable prognostic factors could be treated insufficiently, since the involvement of the axillary lymph nodes is important to indicate the need for postoperative radiotherapy. Ultrasonography has been also recommended to predict axillary lymph node involvement, but ultrasonography features alone are insufficient to replace sentinel lymph node biopsy 1.

To the best of our knowledge, it is the first study that identified predictive factors of axillary lymph node involvement in patients with early invasive breast cancer in the North West of Tunisia. Nevertheless, the main limitations of our study were its retrospective design and the relatively small number of included patients. Besides, the proliferation marker Ki67 and the Her2 status were not mentioned for all cases, since it was not routinely measured and registered in our database at the beginning of the study period.
